# Supplementary material for: Discovery and application of insertion-deletion (INDEL) polymorphisms for QTL mapping of early life-history traits in Atlantic salmon
Source: BMC Genomics. 2010 Mar 8;11:156. doi: 10.1186/1471-2164-11-156 (PMC2838853; doi:10.1186/1471-2164-11-156)
Supplement: Additional file 2 — Information on developed 76 locus single-run INDEL panel in Atlantic salmon. Information on fluorescence labeling, primer concentrations, PCR pooling and links to alignments, INDEL motifs and GENESCAN (Burge and Karlin 1997) predictions of genes/exons are available in html format. [file 1471-2164-11-156-S2.ZIP › Additionalfile2/snpsummary2273.html]

```
Cluster 404 Contig 3

prev  Summary    Contig List  next
```

Size of Consensus sequence = 1763

Number of sequences = 22

Minimum redundancy = 6

Key

A gi|29324431|gb|CB513205.1|CB513205 ssalrgb518058\_rev mixed\_tissue Salmo salar cDNA, mRNA sequence  
B gi|59833795|gb|DN139478.1|DN139478 SGP264318 Atlantic salmon Head kidney cDNA library Salmo salar cDNA clone FN4-2603 5', mRNA sequence  
C gi|85020638|gb|DW549294.1|DW549294 EST\_ssal\_rgb2\_13713 rgb2 Salmo salar cDNA clone ssal\_rgb2\_522\_339\_rev 5', mRNA sequence  
D gi|45320181|gb|CK890448.1|CK890448 SGP150679 Atlantic salmon Ovaries cDNA library Salmo salar cDNA clone KG5-0665 5', mRNA sequence  
E gi|89851665|gb|DY707788.1|DY707788 EST\_ssal\_rgb2\_63527 ssalrgb2 mixed\_tissue Salmo salar cDNA Salmo salar cDNA clone ssal\_rgb2\_602\_228\_rev 5', mRNA sequence  
F gi|85034290|gb|DW562946.1|DW562946 EST\_ssal\_rgb2\_27365 rgb2 Salmo salar cDNA clone ssal\_rgb2\_544\_174\_rev 5', mRNA sequence  
G gi|89858252|gb|DY714375.1|DY714375 EST\_ssal\_rgb2\_70115 ssalrgb2 mixed\_tissue Salmo salar cDNA Salmo salar cDNA clone ssal\_rgb2\_613\_261\_rev 5', mRNA sequence  
H gi|85053357|gb|DW581535.1|DW581535 EST\_ssal\_rgb2\_45954 rgb2 Salmo salar cDNA clone ssal\_rgb2\_574\_126\_rev 5', mRNA sequence  
I gi|45303449|gb|CK873818.1|CK873818 SGP134986 Atlantic salmon Brain cDNA library Salmo salar cDNA clone HJ5-0473 5', mRNA sequence  
J gi|115290503|gb|AM402692.1|AM402692 AM402692 Salmo salar liver Parr Salmo salar cDNA clone liv\_ali\_L3H3, mRNA sequence  
K gi|84990596|gb|DW540946.1|DW540946 EST\_ssal\_rgb2\_5365 rgb2 Salmo salar cDNA clone ssal\_rgb2\_509\_370\_rev 5', mRNA sequence  
L gi|24387219|gb|CA056976.1|CA056976 ssalrgb536330 mixed\_tissue Salmo salar cDNA, mRNA sequence  
M gi|89851666|gb|DY707789.1|DY707789 EST\_ssal\_rgb2\_63528 ssalrgb2 mixed\_tissue Salmo salar cDNA Salmo salar cDNA clone ssal\_rgb2\_602\_228\_fwd 3', mRNA sequence  
N gi|85020639|gb|DW549295.1|DW549295 EST\_ssal\_rgb2\_13714 rgb2 Salmo salar cDNA clone ssal\_rgb2\_522\_339\_fwd 3', mRNA sequence  
O gi|117443931|gb|EG776154.1|EG776154 EST\_ssal\_evd\_51323 ssalevd thymus Salmo salar cDNA Salmo salar cDNA clone ssal\_evd\_569\_056\_fwd 3', mRNA sequence  
P gi|89858253|gb|DY714376.1|DY714376 EST\_ssal\_rgb2\_70116 ssalrgb2 mixed\_tissue Salmo salar cDNA Salmo salar cDNA clone ssal\_rgb2\_613\_261\_fwd 3', mRNA sequence  
Q gi|29318784|gb|CB507138.1|CB507138 ssalob505256 reproductive Salmo salar cDNA, mRNA sequence  
R gi|89884963|gb|DY741086.1|DY741086 EST\_ssal\_rgb2\_96825 ssalrgb2 mixed\_tissue Salmo salar cDNA Salmo salar cDNA clone ssal\_rgb2\_658\_175\_fwd 3', mRNA sequence  
S gi|24389231|gb|CA058988.1|CA058988 ssalrgb518058 mixed\_tissue Salmo salar cDNA, mRNA sequence  
T gi|24390059|gb|CA059816.1|CA059816 ssalrgb533109 mixed\_tissue Salmo salar cDNA, mRNA sequence  
U gi|29325333|gb|CB514107.1|CB514107 ssalrgb548175 mixed\_tissue Salmo salar cDNA, mRNA sequence  
V gi|85034291|gb|DW562947.1|DW562947 EST\_ssal\_rgb2\_27366 rgb2 Salmo salar cDNA clone ssal\_rgb2\_544\_174\_fwd 3', mRNA sequence

3 SNPs detected

A B C D E F G H I J K L M N O P Q R S T U V  cosegregation weighted

739 . . - - G G G G - G - - G - - . - - G . . .   2/3 48.48
740 . . - - T T T T - T - - T - - . - - T . . .   2/3 48.48
741 . . T T T T - T - T - - T T - . - - T . . .   1/3 24.24
